# Unexpected Regularity in Swimming Behavior of *Clausocalanus furcatus* Revealed by a Telecentric 3D Computer Vision System

**DOI:** 10.1371/journal.pone.0067640

**Published:** 2013-06-27

**Authors:** Giuseppe Bianco, Vincenzo Botte, Laurent Dubroca, Maurizio Ribera d’Alcalà, Maria Grazia Mazzocchi

**Affiliations:** Stazione Zoologica Anton Dohrn, Napoli, Italy; Institute of Marine Research, Norway

## Abstract

Planktonic copepods display a large repertoire of motion behaviors in a three-dimensional environment. Two-dimensional video observations demonstrated that the small copepod *Clausocalanus furcatus,* one the most widely distributed calanoids at low to medium latitudes, presented a unique swimming behavior that was continuous and fast and followed notably convoluted trajectories. Furthermore, previous observations indicated that the motion of *C. furcatus* resembled a random process. We characterized the swimming behavior of this species in three-dimensional space using a video system equipped with telecentric lenses, which allow tracking of zooplankton without the distortion errors inherent in common lenses. Our observations revealed unexpected regularities in the behavior of *C. furcatus* that appear primarily in the horizontal plane and could not have been identified in previous observations based on lateral views. Our results indicate that the swimming behavior of *C. furcatus* is based on a limited repertoire of basic kinematic modules but exhibits greater plasticity than previously thought.

## Introduction

In the three-dimensional (3D) aquatic environment, planktonic animals do not simply drift but move to remain in suspension, search for food and mates, and escape predators. Among planktonic copepods, a large variety of behaviors is displayed with evidence of species- and stage-specific swimming patterns [1]–[5]. Numerous attempts to understand the adaptive role of zooplankton swimming behavior have followed the seminal paper by Gerritsen and Strickler [6], which highlighted the importance of swimming speed in determining encounters among planktonic organisms. More recently, the model of Visser and Kiørboe [7] pointed to the role of motion patterns in balancing the probabilities of encountering food items and avoiding predators, thus predicting different optimal swimming strategies to cope with different prey concentrations and distribution. However, differences among the swimming patterns displayed by different species hint at intrinsic, morphological constraints and/or overlooked adaptive traits. Given the behavioral diversity observed in plankton, an in depth analysis and robust characterization of swimming modes will lead to an improved understanding of interactions at the individual level [8].

Many copepod species dwell in large oceanic regions and experience a wide range of environmental conditions, including changes in food quantity and quality. The small copepod *Clausocalanus furcatus* (∼1 mm total length) is among the most common and abundant calanoids in the tropical and subtropical epipelagic waters of the Atlantic Ocean [9]. This calanoid contributes to copepod abundance in the Mediterranean Sea [10] in both open oligotrophic areas [11], [12] and coastal eutrophic waters [13]. This species behaves differently from other copepods analyzed to date. Video observations in two dimensions (2D) of the Atlantic *C. furcatus* showed that adult females move continuously at ∼10 body lengths s^−1^ (BL s^−1^) along convoluted paths occasionally interrupted by sudden somersaults or sinking events [14]. *C. furcatus* captures food particles only from a confined area in front of its head without any evidence of feeding currents or ambush predation [15], which have been observed in other calanoids [16]. The motion parameters and modes of food capture indicate that this species explores small water parcels in rapid succession with a foraging tactic unique among small planktonic copepods; this foraging tactic seems well-suited for rapidly exploiting micro patches of food [14], [15]. Furthermore, on the basis of 2D observations, it has been hypothesized that the motion of *C. furcatus* has random-like properties, which might be a strategy adopted by zooplankton to reduce predation [15].

The distinctive swimming behavior of *C. furcatus* shown by 2D video recording prompted us to use 3D measures to fully analyze movement in real space. For this purpose, we designed and set up a 3D video system with telecentric lenses that allow tracking of zooplankton small-scale behavior without the distortion errors inherent in common lenses.

**Figure 1:**
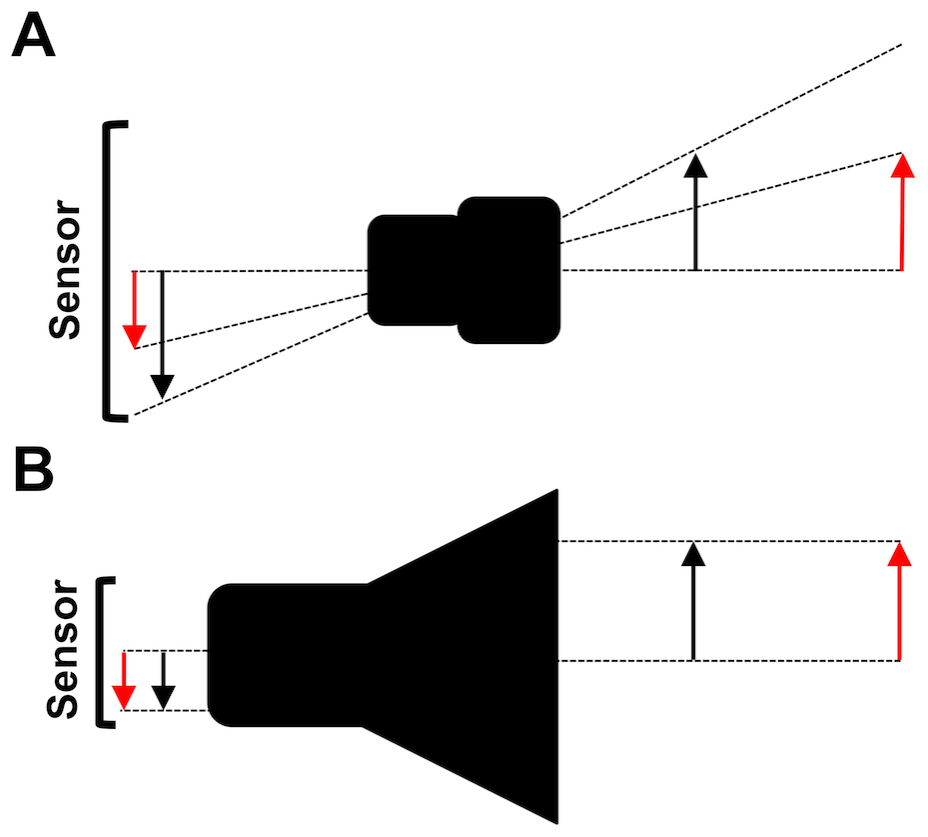
Lens projection comparison. (A) In a standard lens, incoming rays have a conical shape and generate images of different sizes on the sensor when changing the object-to-lens distance. (B) In a telecentric lens, rays enter the optics with a parallel path and generate images of unchanged size on the sensor with object-to-lens distance displacement.

The most common method for observing small-scale movements is the use of two synchronized cameras positioned orthogonally to the aquarium [17]–[19]. The resulting 2D projections of the organism’s position in space are successively processed to recompose the 3D swimming paths. For images captured with common lenses, the perspective of the projection simulates human vision and gives our eyes the illusion of image depth. Conversely, when those lenses are used for precise measurements, the perspective effect introduces uncertainties from the 2D images to the 3D coordinates [20], [21]. For instance, two objects moving at the same speed but at different distances from the camera would cover different amounts of space on the image plane. To avoid measurement errors, it is necessary to apply a camera calibration technique [21], [22], i.e., a mathematical model of how the camera maps the 3D volume on a 2D plane. As an alternative to the camera calibration and for relatively small water volumes (cm-size), an optical system can be implemented that is able to maintain the measurement accuracy over a reasonably long depth of field. This accuracy can be achieved either by collimating the light field [23], [24] or by using telecentric lenses that, given a light source, use only the fraction of light parallel to the optical axis to produce an orthographic view of the objects in the field of view (Fig. 1).

By means of a telecentric 3D computer vision system, we observed unexpected regularities in the swimming behavior of *C. furcatus* that had not been demonstrated previously.

## Materials and Methods

### Ethics Statement

Sampling was performed in accordance with Italian laws for which no specific permissions were required for zooplankton sampling. This study did not investigate endangered or protected species.

### Copepod Sampling and Observations

Zooplankton samples were collected at station LTER-MC (40° 48.5′ N; 14° 15′ E) in the inner Gulf of Naples (Tyrrhenian Sea) on 27 November 2008 and 30 September 2009. Vertical tows were performed gently in the upper 50 m of the water column with a Nansen net (200 µm) equipped with a 5-L glass jar as non-filtering cod-end. Within an hour after collection, the samples were brought to the laboratory in coolers and kept in a room with light cycle and temperature set to the in situ conditions (19–21°C). The settled material was removed from the sampling jars and zooplankton were gently diluted into other glass jars (3–5 L), containing seawater with natural food from the sampling site, to reduce crowding. On the same day and the following morning, *Clausocalanus* individuals were sorted from the jars with a large-bore glass pipette; the genus was identified by size and movement against a fluorescent background light. Each individual was then checked under a dissecting microscope for stage, sex, and species; only healthy, adult, intact females of *C. furcatus* were selected. Adult females were chosen to ensure observation of homogeneous groups of the same species because numerous *Clausocalanus* congeners co-occur in the Gulf of Naples [12], and their copepodites cannot be distinguished at the species level.

**Figure 2:**
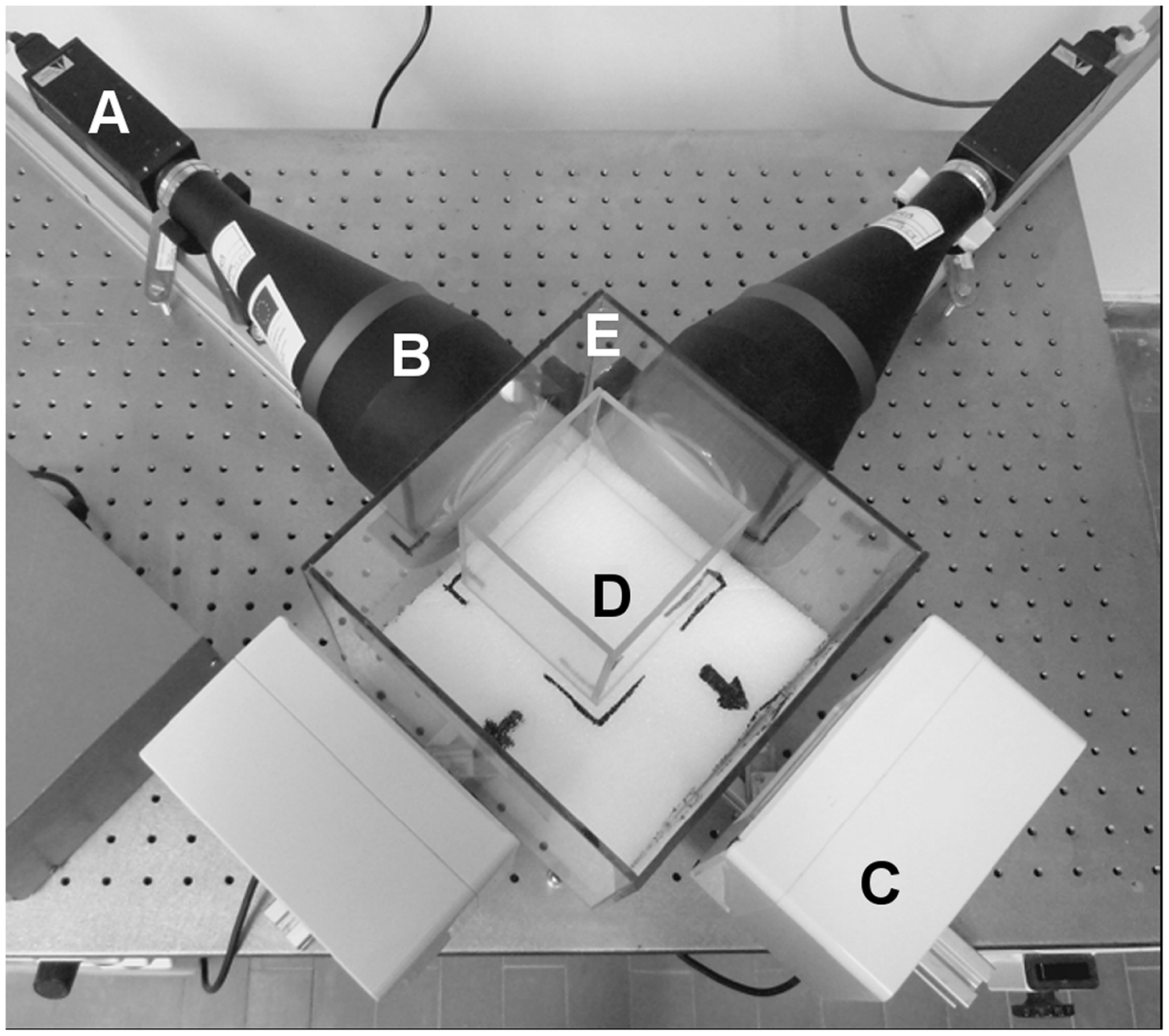
Telecentric stereo vision setup. Two optical-systems were disposed orthogonally and fixed on an anti-vibrating table. Each system was made up of (A) a digital camera, (B) a telecentric lens, and (C) an infrared light source, which were also disposed orthogonally on the anti-vibrating table. The experimental 1-L glass aquarium (D) was placed into an empty 8-L aquarium (E), to be protected from possible external perturbations. Both aquaria were covered with a glass top during recording experiments. The equipment was housed in a temperature-controlled room.

The selected copepods were separated in two homogeneous groups (n = 30–37), which were recorded for one hour, either in the presence of food or without food (Whatman GF/F filtered seawater) for a total of four experiments (Table 1). Before video recording, each group was moved into a 1 L cubic glass aquarium filled with seawater, with or without food depending on the experiment, and left to acclimatize for 15 minutes in the dark. For further protection from external perturbation, the aquarium was placed in a larger (8 L) empty cubic aquarium (Fig. 2). During recording both aquaria were covered by glass tops.

**Table 1:** Experimental conditions and information on the duration of observed trajectories.

			Duration		
Date	Food	Number of trajectories	Cumulative	Maximum	Average
2008	5×10^5^ cells L^−1^	255	1 h 41 min 29 s	4 min 01 s	24 s
	Filtered seawater	384	3 h 04 min 19 s	5 min 42 s	29 s
2009	5×10^6^ cells L^−1^	625	4 h 14 min 04 s	4 min 38 s	24 s
	Filtered seawater	425	4 h 09 min 11 s	4 min 38 s	35 s
**All experiments**		1689	13 h 09 min 03 s	5 min 42 s	28 s

General information on swimming trajectories of *Clausocalanus furcatus* obtained in different years and in the presence and absence of food.

The food consisted of natural particle assemblages collected with Niskin bottles at the surface at the same site and time of zooplankton sampling and gently screened through 200 µm mesh to remove mesozooplankton. In the two successive years, the natural particle assemblage differed only in terms of cell concentration (Table 1). Phytoflagellates <5 µm dominated in both cases; larger cells (>10 µm) contributed less than 15% and were mainly represented by diatoms of the genera *Leptocylindrus* and *Pseudo-nitzschia*. The experiments without food where designed to explore the range of the *C. furcatus* behavioral repertoire. Before recording without food, the selected individuals where kept for two hours in filtered seawater before starting the acclimatization.

### Video Recording Setup

The observational video setup was designed and assembled at the Stazione Zoologica Anton Dohrn (Napoli, Italy) and was based on two identical optical-systems. Each optical-system was composed of a specifically designed telecentric lens (Centre for Advanced Research in Space Optics, Trieste, Italy) with a working focal ratio of 8 that, combined with a ½ inch sensor from a Sony XCD-X700 FireWire digital camera (1024×768 pixel resolution), resolved a field of view of 80×60 mm and a field of depth of ∼ 100 mm. A diffuse infrared (IR) light source composed of an array of four 18 mW, 780 ηm light-emitting diodes powered at 12 V DC allowed video recording under dark condition, which prevented a phototaxic response by the copepods. The two optical-systems were placed at 90° to each other on a VibroPlane 9100 anti-vibrating table (Kinetic System Inc., Boston, USA), and the 1 L aquarium containing the copepods was placed in a central position between the two lenses and the two IR light sources (Fig. 2).

The IR light sources positioned in front of the lenses allowed the cameras sensors to record the silhouette of a free-swimming copepod as a dark array of pixels against a lighter background. The volume of observation, obtained by the intersections of the field of view and depth of field of the two optical-systems, was a parallelepiped of 80×80×60 mm centered in the middle of the aquarium and representing 38% of the aquarium volume. Only copepods that were at least 10 mm away from the walls and 20 mm from the bottom or the top of the aquarium could be recorded. This prevented any interference of the walls on the observed copepod motion. The spatial resolution of the system was 78 µm, and the temporal resolution due to the camera sampling rate was 15 frames s^−1^. The spatial resolution was appropriate for tracking the motion of single copepods and observing their body orientation, although the resolution was not sufficient to show the movements of single body parts or appendages of *C. furcatus* or to see food items and possible capture events.

To test for the telecentricity of the lenses, i.e., that the incoming rays were collimated along the optical axis, and to identify possible effects resulting from the presence of water in the aquarium, solid spheres (15–22 mm diameter) were suspended in the center and in each corner of the aquarium and filmed with and without filtered seawater. Single snapshots were analyzed using ImageJ 1.41 software [25]. The diameter of each sphere was measured, and the results between positions were compared both before and after the addition of seawater. The difference between the measures were always <1 pixel (i.e., <78 µm) and thus compatible with the expected properties of the telecentric lenses combined with illumination by a backlight external to the aquarium. In particular, the effect of the water was negligible because the light rays coming into the aquarium and encountering the air/water interface encounter a successive water/air transition in the opposite direction before reaching the lens, thus annulling any refraction effect. The video equipment was placed in a temperature-controlled room.

### Software and Tracking Analysis

The two digital cameras were controlled and synchronized using custom software (e-voluzione srl, Napoli, Italy) that allowed synchronizing and recording of uncompressed digital videos from both cameras directly onto a PC hard-drive. The videos where processed with a macro script for ImageJ and transformed into binary videos by manually setting a luminosity threshold. For each camera, the 2D swimming trajectories (in the X-Z and Y-Z planes, with Z being the vertical direction) were obtained with the MTrack2 tracking particle plugin [26]. Using a C++ script, the 2D trajectories obtained from the two cameras were merged into 3D trajectories by comparing the common Z values and eliminating ghost trajectories (e.g., non-moving objects). Only trajectories longer than 5 seconds were considered for the present study. For each 3D trajectory, the velocity and the occurrence of sinking events were calculated using custom Java software.

### Analysis of Trajectories and Statistics

For each trajectory, the velocity along the X, Y and Z axes (**u**, **v** and **w**), and the 3D speed *V* were computed for the generic *i*-th point using the central finite difference formula:
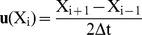
(1)

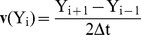
(2)

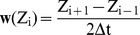
(3)

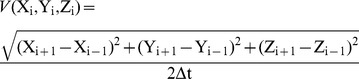
(4)with Δt being the time lag between two consecutive points (1/15 s). The mean swimming speed was obtained by averaging all speed values for each individual trajectory. The active swimming speed was computed excluding any sinking events. For each swimming pattern, the mean speed was calculated as the grand average of the means of all trajectories.

The occurrence of sinking events in a swimming trajectory was identified by an algorithm that takes into account four conditions: (1) downward movement (**w** <0), (2) the vertical direction of displacement (absolute value of **u** and **v** <0.5 mm s^−1^), (3) a swimming speed of *V* <3 mm s^−1^, which preliminary measurements had indicated to be a non-active swimming phase, and (4) a maximum sink duration of 5 s. For each experiment, the percentage of time spent by copepods in sinking was calculated as a fraction of the cumulative time of the full trajectory.

Quality control for trajectory determination was achieved by plotting 3D trajectories taken from different perspectives on the computer and analyzing the trajectories using R 2.9.1 software [27] in combination with the rgl package [28]. The trajectories were carefully checked to (1) exclude trajectories for which errors had been introduced in previous analytical steps, (2) verify that the sinking events were computed correctly, and (3) assign each trajectory to a category according to characteristics of its shape.

The total number of trajectories that occurred simultaneously and that presented the same pattern were counted using an algorithm that searched through each frame of the entire experiment. This procedure provided the number of individuals performing the same pattern simultaneously.

Motion parameters were statistically analyzed using R software. Because the average speed values did not have a normal distribution (Kolmogorov-Smirnov test), the non-parametric Mann-Whitney U-test was used to compare speed between swimming patterns. The Student *t*-test was used to compare swimming activity duration.

### Spectral Analysis

The current understanding of *C. furcatus* swimming, i.e., that swimming patterns are substantially random, derives in part from a spectral analysis applied to 2D trajectories recorded laterally with an instrument closely resembling the CritterCam® [15]. Spectral analysis can be used to evaluate the characteristic periodicities of each trajectory, allowing the identification of peak frequencies associated with the swimming motion. The analysis of Uttieri et al. [15] showed an absence of peak frequencies in velocity.

Along with the approach used by Uttieri et al. [15], velocity as a function of time has been analyzed using the Power Spectral Density (PSD), which describes the data in terms of frequency composition, by applying the fast Fourier transform (FFT) [29] to the velocity data series. The spectral density function represents how the energy associated with the process is distributed over the spectrum of frequencies [30], with the presence of peaks in the spectral density corresponding to characteristic frequencies of motion. In this study, spectral analysis was applied directly to raw velocity data without any filtering or averaging.

**Figure 3:**
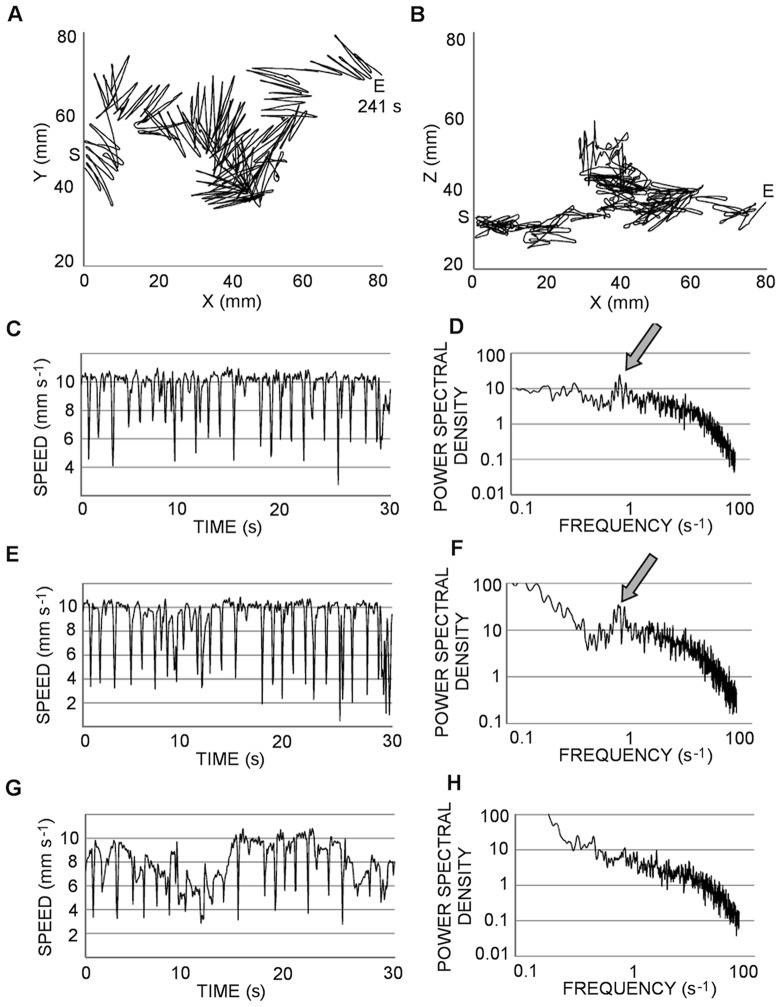
The *Clausocalanus furcatus* high-speed swimming pattern recorded in 2009. The swimming pattern was characterized by sharp turns between straight segments of roughly equal length. Top (A) and lateral (B) views of a 241 s trajectory (S, start; E, end). The three dimensional velocity as a function of time (C) and its power spectrum density (D) are shown for the first 30 s of the trajectory, with the block arrow indicating the dominating frequency in the power spectrum. The successive panels present the two dimensional velocity on the horizontal plane (E) with the corresponding power spectrum density (F), and the velocity on the vertical plane (G) with the corresponding power spectrum density (H). A 1-minute 3D animation of the pattern described in this figure is provided in the supplemental material (Video S1).

The PSD was used to define a characteristic time for each trajectory. If a frequency was clearly dominant in the PSD of the velocity (as in Figure 3D), the inverse of that frequency was taken as the characteristic period of the trajectory.

## Results

From the video recording of *C. furcatus* adult females, 1689 3D trajectories were acquired, with an average duration of 28 s and the longest duration of 5 min 42 s (Table 1). Most trajectories (63–85%) were characterized by three types of remarkably regular patterns composed of segments of similar length connected with recurrent angles (Table 2). The remaining trajectories (15–37%) did not present any regularity and were characterized by consecutive loops interrupted by rare sinking events.

**Table 2:** Occurrence of swimming patterns.

			Maximum number ofsimultaneous trajectories		
Date	Food	Pattern occurrence (% time)	High-speed	Up and sink	Swim and sink
2008	5×10^5^ cells L^−1^	63	2	3	–
	Filtered seawater	76	1	6	1
2009	5×10^6^ cells L^−1^	76	6	5	–
	Filtered seawater	85	2	7	1

Frequency (% of cumulative trajectory duration) of the three patterns observed in *Clausocalanus furcatus* swimming behavior and counts of the greatest number of individuals performing the same pattern simultaneously. Dashes indicate that the pattern was not present.

**Figure 4:**
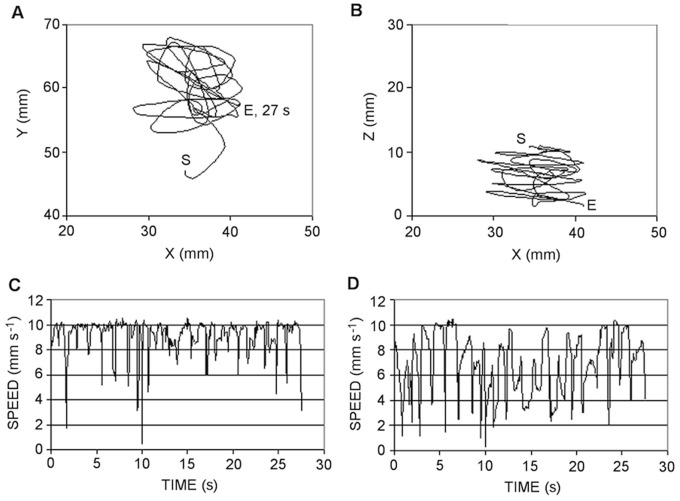
The high-speed swimming pattern. Top (A) and lateral (B) views of a 27 s trajectory recorded in 2008 (S, start; E, end). The successive panels present the two-dimensional velocity on the horizontal plane (C) and on a vertical plane (D).

The first regular pattern frequently observed was a high-speed swimming characterized by a continuous looping, mostly extended on the horizontal plane, rather than the vertical plane, and made of short straight segments (∼ 10 mm) connected by sharp, fast turns (Fig. 3). The copepods moved on planes with very similar inclination (∼ 10°) and repeatedly followed the same pattern in localized clusters. The regularity of this pattern appeared also in the speed diagrams, which showed very fast (>10 mm s^−1^) movements interrupted, at regular time intervals (∼ 1 s), by sharp deceleration when the copepod turned and resumed swimming in the opposite direction (Fig. 3C). The instantaneous speed was significantly higher in the downward, rather than upward, direction (U = 1.6×10^8^, p<0.001). The regularity of this motion was clearly visible in the horizontal plane (Fig. 3A), but disappeared in the vertical plane (Fig. 3B) where the paths looked randomly twisted. There was a regular component to the 3D velocity (Fig. 3C) and the horizontal velocity (Fig. 3E), with corresponding peaks in the PSD (Fig. 3D, F). Conversely, the velocity in the vertical plane (Fig. 3G) presented a less regular pattern, with an absence of a marked peak in the PSD (Fig. 3H) indicating the lack of regularity on that plane. The high values near zero frequency in the PSD (Fig. 3F, H) are clearly spurious, as they did not appear in the three-dimensional velocity. Indeed, it is well known that spectral analysis can produce a spurious bulge of power at low frequencies [29]. The trajectory in Fig. 3 is one of the longest and demonstrates the high-speed pattern. This trajectory was so highly convoluted that the copepod took more than 4 min to cross the entire field of observation (i.e., 80 mm linear distance) (see also Video S1). This pattern was performed by up to 6 individuals simultaneously and was recorded both in the presence and absence of food and in both years (Table 2, Figs. 3, 4).

**Figure 5:**
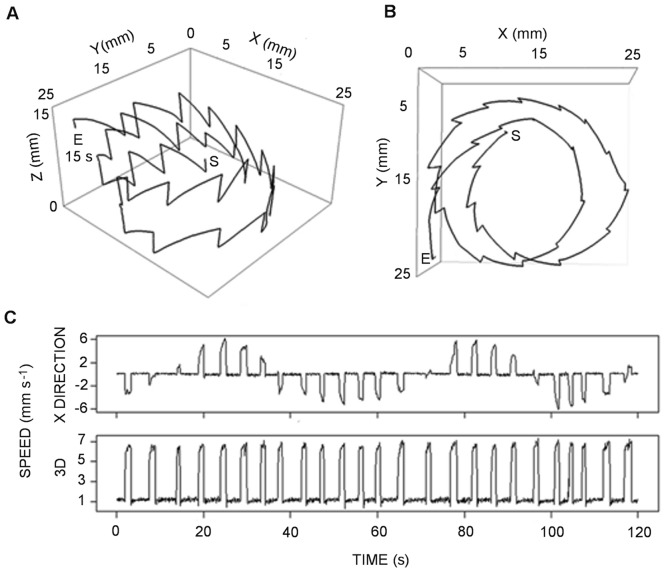
Example of the *Clausocalanus furcatus* up and sink swimming pattern. Lateral (A) and top (B) views of a 120 s trajectory (S, start; E, end), and corresponding diagrams of the velocity along the X-axis direction and of the 3D speed (C). A 1-minute 3D animation of the pattern described in this figure is provided in the supplemental material (Video S2).

The second motion behavior frequently observed consisted of an up and sink pattern, characterized by a regular alternation between swimming upward and sinking (Fig. 5A). After each sinking phase, individuals swam upward just enough to return to the previous depth level (Video S2). At the same time, the copepod proceeded along a regular horizontal circle, which was clearly visible from the top view of the trajectory (Fig. 5B). Two different periodicities could be observed in the velocity diagrams: the repetition (every 5 s) of the upward (6.0±1.5 mm s^−1^) and sinking (1.2±0.2 mm s^−1^) movements and the sinusoidal signal along one horizontal axis alternate to the sinking phases (Fig. 5C). The active swimming lasted for a significantly shorter time (*t*-test, p<0.001) than the sinking phase (1.4±1.0 s and 4.0±2.1 s, respectively). As a result, this pattern was performed at a very low average speed (2.4±0.8 mm s^−1^) and was the slowest pattern recorded (Table 3). This pattern was observed in several individuals simultaneously, both in the presence and absence of food and in both years (Table 2).

**Table 3:** Speed and duration of swimming patterns.

	Speed ± σ (mm s^−1^)			Duration ± σ (s)	
Pattern	Active	Sinking	Total	Swimming	Sinking
High-speed	–	–	9.9±1.4	–	–
Up and sink	6.0±1.5	1.2±0.2	2.4±0.8	1.4±1.0	4.0±2.1
Swim and sink	5.2±0.5	1.0±0.5	3.0±0.5	2.9±1.2	3.0±0.6

Swimming performance metrics of the three swimming patterns observed in *Clausocalanus furcatus.* Swimming speed was computed using the instantaneous speed values of the swimming phases (Active), passive swimming (Sinking), and both phases taken together (Total). Sinking was not observed as part of the high-speed pattern.

**Figure 6:**
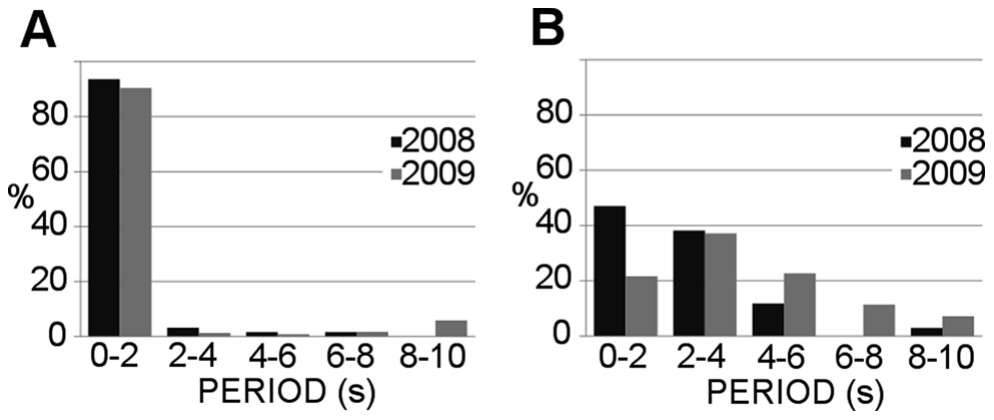
Comparison of the periods obtained from the power spectrum analysis. The histograms compare the distributions of the characteristic periods obtained using the Power Spectrum Density analysis of the velocity module as a function of time separately for high-speed swimming patterns (A) and up and sink patterns (B), in the two successive years.

**Figure 7:**
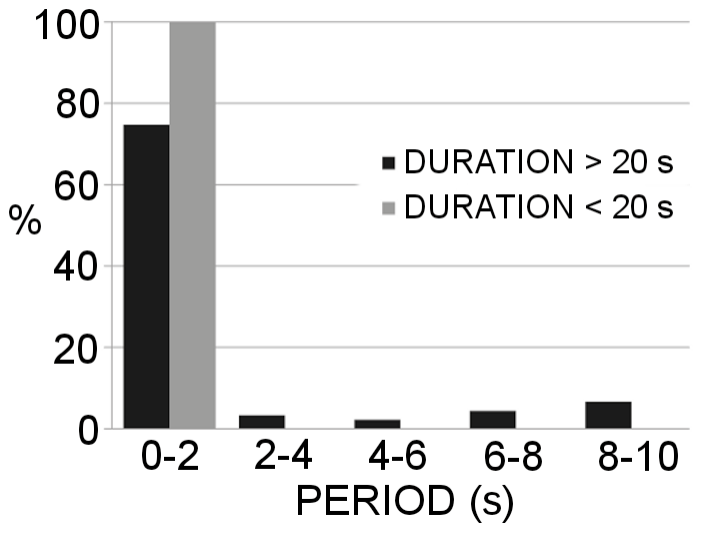
Comparison of the periods obtained for short and long trajectories. The Power Spectrum Density analysis applied to the velocity module as a function of time of all trajectories recorded in 2009, shown separately for trajectories with durations either longer and shorter than 20 s.

The PSD showed clear differences in the periods characterizing the high-speed swimming and the up and sink patterns, but was remarkably consistent between the two years of observation (Fig. 6). The high-speed swimming trajectories had a characteristic period of only 0–2 s (Fig. 6A), while the up and sink trajectories were more variable, occurring primarily for 0–2 s or 2–4 s (Fig. 6B). Overall, regular peaks in the PSD were recorded in ∼70% of the trajectories. The characteristic periodicity of trajectories was independent of their duration because it was recorded in both <20 s and >20 s trajectories (Fig. 7). Several trajectories (data not shown) displayed a transition between these two different patterns, indicating that an individual was able to switch from one swimming mode to the other.

**Figure 8:**
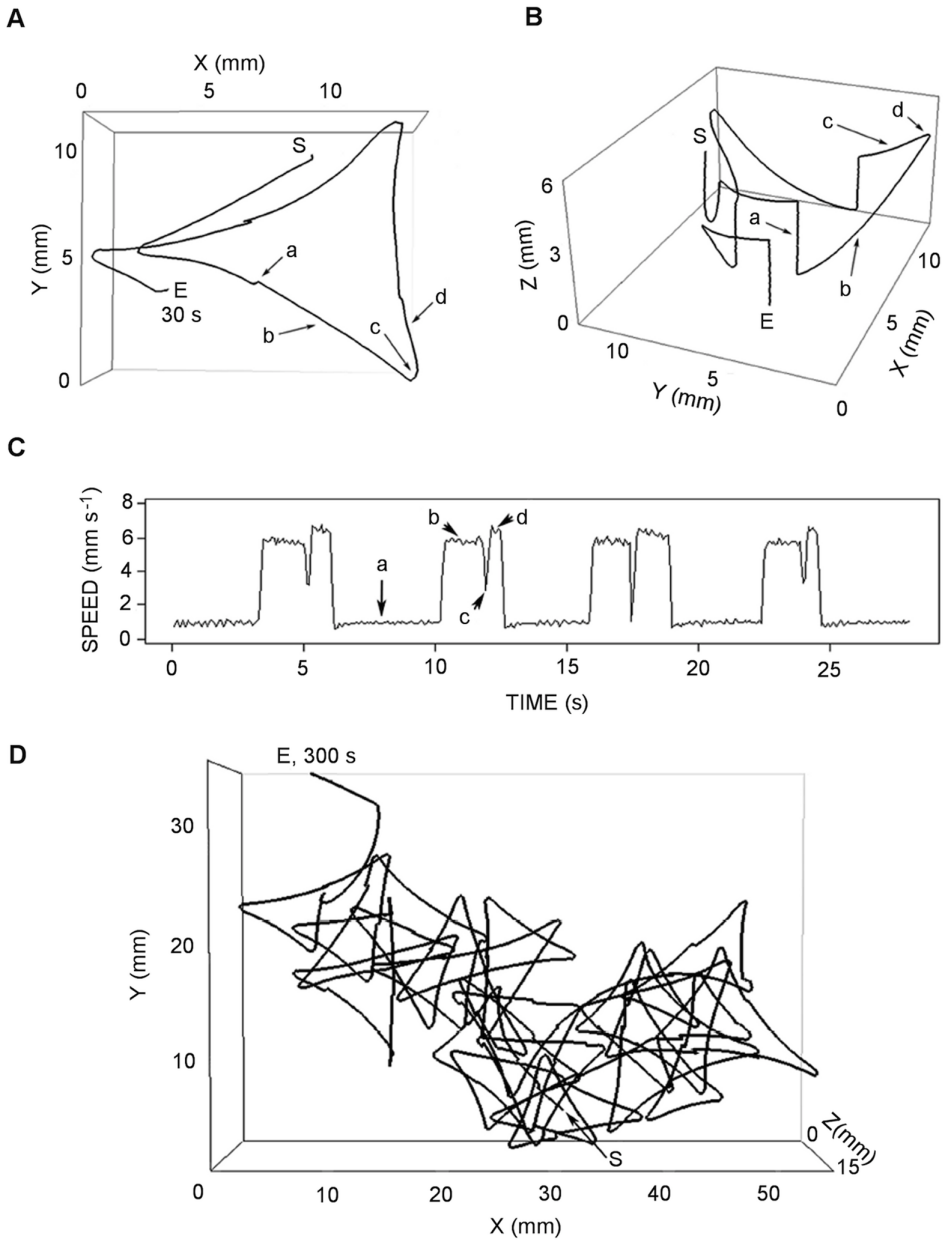
Example of *Clausocalanus furcatus* swim and sink motion pattern. This type of pattern was recorded in the absence of food (in filtered seawater) both in 2008 and 2009. Top (A) and lateral (B) view of a 30 s trajectory (S, start; E, end) and corresponding velocity diagram (C). The pattern is composed by a sequence of (a) sinking, (b) upward swimming, (c) ample turning, and (d) downward swimming. Top view (D) of a 5 min long trajectory showing the same pattern. A 1-minute 3D animation of the pattern described in this figure is provided in the supplemental material (Video S3).

The third type of behavior was a swim and sink pattern and was observed only in the absence of food. In this case, the swimming motion consisted of a sequence of sinking, swimming upward, fast turning, and swimming downward (Fig. 8; Video S3). The different phases of the motion were travelled at different speeds, as shown by the speed diagram (Fig. 8C). After sinking at ∼ 1 mm s^−1^, the copepod swam upward (∼ 6 mm s^−1^), turned at lower speed (∼ 3 mm s^−1^), swam downward at higher speed (∼7 mm s^−1^), and then sunk before resuming the same sequence (Fig. 8C). The repetition of this pattern described another larger trajectory, which appeared, from the top view, similar to a series of open triangles that spanned large partially overlapping areas (Fig. 8D).

The swim and sink pattern was observed only in experiments without food and only by one individual at a time (Table 2). It is possible that the behavior was always exhibited by the same individual within a group; however, it is worth noting that the pattern appeared in experiments performed by individuals in different populations in two successive years (Table 2).

The comparison among the three regular patterns described above (Table 3) shows that swimming was significantly faster (U = 2.8×10^4^, p<0.001) and lasted significantly longer (*t*-test, p<0.001) in the swim and sink than in the up and sink patterns. However, the speed in the former pattern was three times slower (U = 2.7×10^4^, p<0.001) than in the high-speed swimming pattern.

## Discussion

### Relevance of the Telecentric Video Recording Set Up

Our understanding of zooplankton small-scale behavior has notably improved in recent years due to the progress in observational methods. Advances in video camera performance and computer vision algorithms have made it possible to reach greater levels of accuracy in reconstructing swimming patterns and measuring motion activity of single individuals. For small animals that live and perform in the water column, only 3D observations allow swimming patterns to be described precisely [31], thus providing the correct positions and speed measurements [21]. However, 3D observations require a more complex experimental system. In particular, accurate camera calibration is needed to avoid the perspective and distortion effect of common lenses [20], [22]. A system equipped with collimated light or telecentric lenses requires simpler computational procedure [32] and is less prone to measurement errors during the stereo reconstruction process [21], due to the simpler optical geometry [20], [33]. With our telecentric system, we discovered new components in the swimming patterns of the planktonic copepod *C. furcatus.*

Our study reveals remarkable and unexpected regularities in 3D trajectories, such as the repetition of geometrical modules that create larger-scale regular patterns, e.g., circles or triangles. These results are in contrast to those of Uttieri et al. [15], whose PSD analysis of high-speed swimming in females showed an absence of peaks. The reason for such a discrepancy is, in our opinion, twofold. First, in the case of the high-speed pattern, the regularity appeared primarily in the horizontal plane and disappeared when the convoluted paths were projected in a 2D lateral vision, where they appeared randomly entangled (Fig. 3). The analysis by Uttieri et al. [15] was based on data derived from a lateral view of the trajectories and was therefore unable to capture the properties of the horizontal patterns. Second, additional discrepancy may originate from the differences in the optical systems used. Telecentric lenses allow the determination of the exact position of the organism at any distance from the lenses, while other systems might introduce position errors that vary with distance [20], [21].

### How Behavioral Modes Relate to Specific Traits

In the presence of natural particle assemblages, as would be found in eutrophic coastal waters, *C. furcatus* was very active most of the time, moving faster and sinking less at higher food concentrations. This calanoid moves at approximately 10 BL s^−1^, a speed recorded both in Mediterranean (present study) and Atlantic populations [14], [15]. *C. furcatus* is much faster than the other marine calanoid females investigated to date, e.g., *Acartia tonsa* (∼4 BL s^−1^ [34]), *Centropages hamatus* (∼6**BL s^−1^ [35]), *Centropages typicus* (∼1 BL s^−1^ [35]), *Centropages velificatus* (∼1.6 BL s^−1^ [36]), *Paracalanus parvus* (∼0.7 BL s^−1^ [35]), *Pseudocalanus elongatus* (∼0.4 BL s^−1^ [35]), *Pseudocalanus minutus* (∼1.2 BL s^−1^ [37]), *Temora longicornis* (∼5 BL s^−1^ [1]).

Independent of the shape of the trajectories, *C. furcatus* swam with a very regular stroke, which might be due to morphological constraints inherent to the body structure. The speed changed only according to the vertical direction, i.e., speed was always slower when the copepod moved upward and faster when it moved actively downward, highlighting the effect of gravity, which affects most zooplankton species [38]. The anisotropy of the space as felt by the copepod, likely due to gravity, was also apparent in the different trajectories in the XY plane vs. XZ and YZ planes as mentioned above. We never recorded females or juveniles of *C. furcatus* moving linearly for more than 15 mm, indicating strict constraints on the behavioral performance of this species and a likely inability to explore longer horizontal distances.

The tendency of *C. furcatus* to remain confined to a restricted area was also shown by the low values of fractal dimension of the 2D trajectories [15] and by a modeling approach [39], which suggested that the copepods sequentially explore neighboring small volumes of water. This behavior has been interpreted as a profitable strategy for exploiting patches of food at the micro-scale [15]. Conversely, *C. furcatus* has a low ingestion rate (on the order of 10^2^–10^3^ particles per day) both in the field [40] and in the laboratory [41], indicating that the number of particle captures is very low during most of the time spent in swimming. Considering the low selectivity in prey capture [40], the low ingestion rate suggests that continuous swimming is not necessarily aimed at increasing ingestion, independently of food concentration. A possible explanation for such convoluted motion could be a defense mechanism against certain predators. This mechanism would explain the low predatory rates by chaetognaths on *C. furcatus* [42] and the negligible occurrence of this species in the guts of *Sagitta* spp. during a 3-year investigation at station LTER-MC (P. Simonelli and M. G. Mazzocchi, unpublished data).

In the absence of food, *C. furcatus* spent most of the time sinking, with an upward relocation at low speed. Absence of food is an unrealistic condition because even in the oligotrophic subtropical Atlantic particle density is on the order of 10^1^ cells ml^−1^ [40], but the no food scenario reveals an intrinsic behavior that is not driven by external signals produced by the presence of food particles. This behavior clearly involves the lowest energy expenditure among the observed swimming behaviors and suggests that *C. furcatus* tends to save energy by keeping disturbance as low as possible, with a motion that would drastically reduce the encounter with both active-searching and ambush predators [7], [8]. Direct measurements of oxygen consumption demonstrated that the type of motion does affect energy expenditures of planktonic copepods [43]. The fast continuous moving *C. furcatus* consumes 2-fold as much oxygen (in relation to the ash-free body weight at 20°C) as does the slow cruising *Paracalanus aculeatus*, and oxygen consumption is even greater compared with the occasionally moving *Oncaea* spp. [43].

Laboratory experiments showed that *C. furcatus* had enhanced reproductive rates and longevity at lower rather than higher food concentrations, indicating a clear adaptation to low food environments [41]. The maintenance of similar peak abundance in both oligotrophic (∼ 85 females m^−3^ [12]) and eutrophic conditions (72 females m^−3^ averaged in 1984–2010 at station LTER-MC in the inner Gulf of Naples, M.G. Mazzocchi and I. Di Capua, unpublished data) further supports a life history trait rather than a resource constraint in the numerical growth of *C. furcatus* populations.

### Ecological Implications of *C. furcatus* Behavioral Plasticity

The first observations of *C. furcatus* swimming behavior, based on a limited number of 2D trajectories recorded in the presence of cultured phytoplankton, suggested that adult females do not visibly change patterns of motion under different environmental conditions [14]. Conversely, the present analysis of 3D trajectories showed that this species is more flexible than previously thought. In the presence of natural food particle assemblages, *C. furcatus* was able to switch from a fast (though spatially limited) explorative swimming to a resting phase with only relocation. In the absence of food particles, the sequence of sinking, upward swimming, turning, and downward swimming seems to represent an intermediate solution between attempting to search for feeding opportunities while also saving energy.

Behavioral plasticity was observed in *C. furcatus*, despite a limited repertoire of regular swimming modules. We hypothesize that these behaviors may be produced by simple neural mechanisms associated with a clear perception of the surroundings, as revealed by the emerging higher-level patterns, most likely based on gravity force sensing. Additional research into *C. furcatus* neurophysiology is necessary to understand these behavioral adaptations fully and to illuminate the relative importance of ultimate vs. proximate factors in determining the success of *C. furcatus* populations.

## Supporting Information

Video S1 (MOV)

Video S2 (MOV)

Video S3 (MOV)
